# Fluid‐preserved fishes are one solution for assessing historical change in fish trophic level

**DOI:** 10.1002/ece3.7061

**Published:** 2020-11-25

**Authors:** Rachel L. Welicky, Terry Rolfe, Karrin Leazer, Katherine P. Maslenikov, Luke Tornabene, Gordon W. Holtgrieve, Chelsea L. Wood

**Affiliations:** ^1^ School of Aquatic and Fishery Sciences University of Washington Seattle WA USA; ^2^ Unit for Environmental Sciences and Management North–West University Potchefstroom South Africa; ^3^ School of Oceanography University of Washington Seattle WA USA; ^4^ Burke Museum of Natural History and Culture University of Washington Seattle WA USA

**Keywords:** compound‐specific stable isotope analysis, glutamic acid, historical ecology, natural history collections, phenylalanine, trophic ecology

## Abstract

1. There are few resources available for assessing historical change in fish trophic dynamics, but specimens held in natural history collections could serve as this resource. In contemporary trophic ecology studies, trophic and source information can be obtained from compound‐specific stable isotope analysis of amino acids of nitrogen (CSIA‐AA‐N).
2. We subjected whole *Sebastes ruberrimus* and *Clupea pallasii* to formalin fixation and 70% ethanol preservation. We extracted tissue samples from each fish pre‐fixation, after each chemical change, and then in doubling time for 32–64 days once placed in the final preservative. All samples were subjected to CSIA‐AA‐N, and their glutamic acid and phenylalanine profiles and associated trophic position were examined for differences over time by species.
3. Glutamic acid and phenylalanine values were inconsistent in direction and magnitude, particularly during formalin fixation, but stabilized similarly (in 70% ethanol) among conspecifics. In some cases, the amino acid values of our final samples were significantly different than our initial pre‐preservation samples. Nonetheless, significant differences in glutamic acid, phenylalanine, and estimated trophic position were not detected among samples that were in 70% ethanol for >24 hr.
4. Our results suggest that the relative trophic position of fluid‐preserved specimens can be estimated using CSIA‐AA‐N, and CSIA‐AA‐N estimates for fluid‐preserved specimens should only be reported as relative differences. Timelines of trophic position change can be developed by comparing specimens collected at different points in time, revealing trophic information of the past and cryptic ecosystem responses.

## Graphical abstract

How can we detect historical change in fish trophic position? We can use compound specific stable nitrogen isotope analysis of fluid‐preserved museum specimens. Due to preservation effects, we should compare similarly preserved fish by species and report relative change in trophic position.

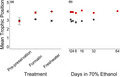

## 1 INTRODUCTION

Historical ecology is an emerging field that uses unconventional forms of ecological data to acquire information on ecosystems of the recent past (McClenachan et al., 2012, 2015; Szabó & Hédl, 2011). Such unlikely data sources as family photos (e.g., Rohde & Hoffman, 2012), maps denoting vessel movements (e.g., McClenachan et al., 2017), fishery catch records (e.g., Bom et al., 2020; Rosenberg et al., 2005), and restaurant menus (e.g., Van Houtan et al., 2013) have all been used to gain insight into environmental dynamics that were previously poorly understood. Historical ecology has allowed for substantial advancement in our understanding of long‐term ecological processes and ecosystem responses to global change (e.g., Sagarin & Micheli, 2001).

One promising but underutilized source of historical data has been hiding in plain sight (Harmon et al., 2019; Holmes et al., 2016): specimens held in natural history collections. Specimens themselves contain ample information on the ecosystems from which they were originally collected. And, museums retain field notes that often include exact catch dates and details about the collection location. Recently, dry bee specimens of entomological natural history collections dating from the 19th and 20th centuries helped resolve some of the mechanisms leading to the decline of bees. By quantifying pollen type and load from the external appendages of bee specimens, researchers were able to determine the historic preferences of these bees for different plants. They were then able to associate these preferences with a decline in specific plant species (Scheper et al., 2014). An important feature of this work is that the preservation process did not alter the integrity of the specimens in a manner that constrains the inferences that can be made by examination of those specimens.

Food web dynamics are the fundamental processes of ecology, and yet these interactions are difficult to reconstruct for historical ecosystems. In contemporary ecosystems, trophic ecology studies frequently use bulk stable nitrogen and carbon isotope analysis of an organism's tissues to track dietary, metabolic, and environmental patterns, as well as the overall trophic position of organisms (e.g., Pool et al., 2017; Welicky et al., 2017). An increasing number of studies are examining historical change in trophic interactions using non‐fluid‐preserved natural history collection specimens (e.g., Blight et al., 2015; English et al., 2018; Jaeger & Cherel, 2011). However, fluid‐preserved specimens are also extremely abundant in natural history collections; for example, there are nearly 8 million fluid‐preserved fish specimens in just four large natural history collections (Harmon et al., 2019).

Does fluid preservation render specimens useless for isotope analysis? Preservation does involve a major chemical restructuring of the specimen, as whole specimens accessioned into natural history collections are typically fixed in formalin and then stored in ethanol. Fixative can alter nitrogenous bonds via cross‐linking, changing protein structures, and creating new products (e.g., Hoffman et al., 2015; Sutherland et al., 2008). Experimental preservation studies report both the enrichment and depletion of bulk stable carbon and nitrogen isotope values of fish tissues and use an array of preservation protocols, making results difficult to compare (e.g., Arrington & Winemiller, 2002; González‐Bergonzoni et al., 2015; Sarakinos et al., 2002). Additionally, fluid‐preserved specimens are not often preserved in the same manner as samples that represent their environmental baselines (e.g., botanical specimens). Specimens that have undergone different chemical processes for preservation are likely not comparable for stable isotope analysis. Therefore, linking primary producer data with fluid‐preserved specimens is exceedingly difficult.

In the past few years, a new kind of analysis—compound‐specific stable isotope analysis of amino acids (CSIA‐AA)—has become available to ecologists and could unlock the trophic information contained in fluid‐preserved natural history collections (Whiteman et al., 2019). Whereas bulk isotopic analysis quantifies a mean isotopic composition off all nitrogen in tissue, CSIA‐AA quantifies the isotopic composition of nitrogen in individual amino acids. The increased resolution and detail provided by these more highly resolved data address several key issues that limit the use of bulk stable isotope analysis (SIA) of fluid‐preserved specimens (McClelland & Montoya, 2002; McMahon & McCarthy, 2016). Compared to SIA, CSIA‐AA environmental information is integrated into individual samples. Accordingly, baseline variation is removed and separate primary production references to give specimens environmental context are not necessary (e.g., Bowes & Thorp, 2015; Chikaraishi et al., 2014). For CSIA‐AA, amino acids are commonly subdivided into two groups: trophic and source. Trophic amino acids (alanine, aspartic acid, glutamic acid, valine, isoleucine, leucine, and proline) are those that have large stepwise enrichments resulting from carbon–nitrogen bonds being cleaved during the metabolism of nitrogenous materials, such that they undergo extensive transamination (Chikaraishi et al., 2014; O'Connell, 2017; Popp et al., 2007). Accordingly, trophic amino acid values provide insight into an organism's diet and metabolic processes. Other amino acids are considered source amino acids (phenylalanine, methionine, glycine, serine, and tyrosine) because they have small enrichments due to less nitrogenous material being cleaved during metabolism, such that there is little transamination. Therefore, the source amino acids aid in inferring an organism's diet and reflect environmental and primary production information (e.g., Chikaraishi et al., 2009, 2010; O'Connell, 2017). By examining the difference in the trophic and source amino acid data of an individual, and examining this difference in relation to taxon‐specific constants, the trophic level of an organism can be determined with a higher degree of resolution than can be achieved via SIA (e.g., Chikaraishi et al., 2014; Choy et al., 2012; Lorrain et al., 2015).

Recently, four papers explored the effects of preservatives on the reliability of CSIA‐AA‐derived trophic‐level estimates. Their preservation methodologies were diverse, and in some cases, the samples they preserved were unlike the specimens of natural history collections. Ogawa et al. (2013) examined the effects of preservation on compound‐specific stable isotope analysis of amino acids of nitrogen (CSIA‐AA‐N) values of fish by experimentally fixing fish muscle tissue in 5% formalin; however, most natural history collections fix specimens in 10% formalin and then store them in 70% ethanol (e.g., Simmons, 2014). More recently, several studies have examined the effects of preservation on fish tissue. They all used 10% formalin and then exposed their fish tissues to this chemical for different lengths of time (48 hr, Hetherington et al., 2019; 1 week, Chua et al., 2020; 4 weeks, Durante et al., 2020). After formalin fixation, some studies subjected their samples to a freshwater bath but immersion time varied (48 hr, Hetherington et al., 2019; 24hr, Chua et al., 2020; not detailed or omitted, Durante et al., 2020). Lastly, samples were preserved in different ethanol concentrations (95% ethanol, Hetherington et al., 2019; 70% ethanol, Chua et al., 2020; graduated from 50% to 70% ethanol over 3 weeks, Durante et al., 2020). Given these studies use various methods, cross‐study comparison is inherently difficult. Interestingly, Chua et al. (2020) and Hetherington et al. (2019) experimentally fixed small muscle samples of fish rather than whole fish; whole fish are typically preserved in natural history collections. The amount and type of tissue preserved could influence the effects of fixation. The rate of chemical penetration increases as surface area to volume of a specimen increases, and because large specimens will exchange more fluid and different types of fluid with surrounding preservative than small specimens, this could change the concentration and composition of chemicals in a fixing jar (Simmons, 2014). Notably, these studies took before and after measurements, providing data to determine if a change occurred. To capture when changes stop, and the rate at which the effects of preservation occur and stabilize, more time points are needed.

The purpose of this study was to test whether CSIA‐AA‐N can be used to generate reliable trophic‐level estimates for fish that have been fixed using the fluid‐preservation method most commonly employed in natural history collections (e.g., Jenkins et al., 2014). We sought to understand whether the estimated trophic positions of fluid‐preserved specimens differed from pre‐fixation samples and to understand how this varied across time as specimens were passed through the preservation process. We specifically wanted to examine the effect of preservation time on ^15^N:^14^N such that we could identify whether specimens preserved for different amounts of time were comparable. In this study, we address three questions: (1) What is the magnitude of change in glutamic acid and phenylalanine profiles over the course of the preservation process? (2) Do these changes stabilize, and if so when does stabilization occur? and (3) Do the trophic position values calculated from glutamic acid and phenylalanine differ over the course of preservation?

## 2 METHODS

### 2.1 Preservation and sampling of fish specimens

We sourced whole, intact, frozen Yelloweye Rockfish (*Sebastes ruberrimus*; *n* = 4; mean total length ± *SD* = 52.50 ± 6.12 cm, mean mass ± *SD* = 2.67 ± 0.91 kg) and Pacific Herring (*Clupea pallasii*; *n* = 5, mean total length ± *SD* = 10.42 ± 0.13 cm, mean mass ± *SD* = 8.11 g ± 0.40 g) for this study. We chose these two species because they have vastly different lipid composition and trophic positions (Yelloweye Rockfish, trophic level = 4.4; Pacific Herring, trophic level = 3.2; reported from fishbase.org). Each fish was subjected to the fluid‐preservation protocol used in the Ichthyology Collection of the University of Washington's Burke Museum of Natural History and Culture (UWFC), which is one of the largest fish collections in North America. Prior to submersion in the first chemical treatment, a pre‐preservation sample, the control/frozen tissue sample, was extracted from under the dorsal fin and was frozen at −20°C for later analysis. Then, in accordance with UWFC protocols, a ~5‐cm incision was made ventrally in each Yelloweye Rockfish to allow chemical fixatives to enter the body cavity and organs of these large specimens. Each specimen was individually housed and submerged in 10% formalin (3.7% formaldehyde) solution for 2 weeks (for Yelloweye Rockfish) and 1 week (for Pacific Herring). After this treatment, another tissue sample was extracted from each specimen and the containers were rinsed with freshwater. The containers were then filled with freshwater and the specimens were submerged for 24 hr. After 24 hr, the water was exchanged with new freshwater and the specimens were again submerged for 24 hr. Following the 48 hr freshwater bath, a tissue sample was extracted from each fish, and then, the fish were placed in their final chemical fixative, 70% ethanol. Tissue samples were then extracted after 24 hr in 70% ethanol and then in doubling time, such that tissue samples were taken at day 1, 2, 4, 8, 16, and 32 for Pacific Herring and at day 1, 2, 4, 8, 16, 32, and 64 for Yelloweye Rockfish. All tissue samples were stored frozen at −20°C until preparation for compound‐specific stable nitrogen isotope analysis.

### 2.2 Compound‐specific stable nitrogen isotope analysis

#### 2.2.1 Amino acid derivatization

We extracted and derivatized amino acids from each fish tissue sample using a modified acetyl chloride‐pivaloyl chloride derivatization process based on Chikaraishi et al. (2009), Metges et al. (1996), and Popp et al. (2007). A standard amino acid mixture (including glutamic acid and phenylalanine) was also prepared. The aforementioned procedures are detailed in Appendix S1.

#### 2.2.2 Isotopic analysis

Automated sampling of amino acid derivatives was conducted using a Trace 1310 GC (Thermo Scientific) in combination with a TriPlus RSH autosampler (Thermo Scientific). A sample volume of 0.1 μl was injected into an injection port held at 240°C in splitless mode onto a capillary column (DB‐35, 30 m × 0.320 mm ID × 0.50 μm film thickness, Agilent J&W GC Columns). The GC oven temperature program for a single injection is detailed in Appendix S2.

All eluting compounds off the column were oxidized inside a GC Isolink II (Thermo Scientific) combustion interface containing a reactor held at 1,000°C. Water was removed through a Nafion membrane downstream of the reactor while CO_2_ was cryogenically trapped in tubing submerged in liquid nitrogen before transfer to the IRMS (DELTA V; Thermo Scientific) through a Conflo IV (Thermo Scientific) universal interface.

High purity N_2_ (>99.9997% N_2_, Airgas) was used as reference gas to initially calculate the isotopic composition. Raw data were drift‐corrected (for drift correction procedure see Appendix S3). Average precision across all amino acids was 0.33‰. Individual precision of each amino acid is given in Appendix S4.

### 2.3 Statistical analysis

#### 2.3.1 Detecting change in glutamic acid and phenylalanine profiles over the course of the preservation process

We were interested in quantifying how the trophic and source amino acid values varied during each chemical change of fixation and preservation and between each time point once specimens were placed in 70% ethanol. We specifically were interested in examining glutamic acid and phenylalanine because these are the trophic and source canonical amino acids most commonly used in the calculation of an organism's trophic position (e.g., Brault et al., 2019; McMahon & McCarthy, 2016; Nielsen et al., 2015). Data for glutamic acid, phenylalanine, and all other amino acids are given in Appendix S5. Therefore, we examined the δ^15^N value differences among each of these amino acid values for each time point by species, using four generalized linear models (Equation 1). Models were selected based on best fit and lowest Akaike information criterion value (AIC). For the response variable, δ^15^N_GLU_, models were implemented with a Gaussian distribution with a link identity function, because Yelloweye Rockfish data were normally distributed. Pacific Herring data were near normal in shape, such that when Gaussian, inverse Gaussian, and gamma models were competed, the Gaussian distribution (with a link identity function) produced the best fit and lowest AIC. The δ^15^N_PHE_ of Yelloweye Rockfish and Pacific Herring was fitted with a gamma and inverse Gaussian distribution, respectively, after these models were evaluated for best fit. Both gamma and inverse Gaussian distributions are commonly applied to data where large outcomes can occur even when small outcomes are more common, and these models produce a peak near the onset and then diffuse toward a natural minimum of 0 and slope of 0. All models were implemented in base R using the *glm()* function. Tukey pairwise comparisons of sampling times were determined using the *ghlt()* function from the package, multcomp v1.4‐12 (Hothorn et al., 2008). We report both the raw *p*‐values of this test as well as those adjusted for a false discovery rate (Benjamini & Hochberg, 1995).

(1) $$\delta^{15}N_{\text{AA}}∼\text{Treatment}$$

#### 2.3.2 Detecting the rate of change in glutamic acid and phenylalanine over the time course of ethanol preservation

We were interested in determining when, during the time in which the specimens were in 70% ethanol, changes in the values of δ^15^N_GLU_ and δ^15^N_PHE_ from one time point to the next became negligible. Therefore, we calculated the difference in amino acid values between time points in relation to the number of days they were in 70% ethanol. Using these values, we examined the relationship between the difference in the values of δ^15^N_GLU_ or δ^15^N_PHE_ and the difference in days using generalized linear models (Equation 2). For the Yelloweye Rockfish, the glutamic acid and phenylalanine models were fit with gamma and Gaussian distributions, respectively, and for the Pacific Herring, the glutamic acid and phenylalanine models were fit with Gaussian and inverse Gaussian distributions, respectively (see above for an explanation of model selection). Models were implemented and pairwise comparisons were made using the aforementioned approaches, packages, and functions.

(2) $$(\Delta \delta^{15}N_{\text{AA}}/\Delta \;\text{number}\;\text{of}\;\text{days}\;\text{in}\;70\%\;\text{ethanol})∼\text{Treatment}$$

#### 2.3.3 Detecting changes in estimated trophic position over the course of preservation

Ultimately, we sought to determine whether chemical preservation significantly influenced the estimated trophic position value of preserved fish. Using the δ^15^N_GLU_ and δ^15^N_PHE_ values from each sampling bout, we quantified each sample's trophic position using the formula for calculating trophic position from Chikaraishi et al. (2009, 2014) (Equation 3). We then tested whether these trophic position values were significantly different among samples by species using generalized linear models (Equation 4) with Gaussian distributions, as they produced the best fit and lowest AIC during model selection. Models were selected and implemented and pairwise comparisons were made using the aforementioned approaches.

(3) $$\text{Trophic position}=\left[\left(\left(\left(\delta^{15}N_{\text{GLU}}‐\delta^{15}N_{\text{PHE}}\right)‐3.4‰\right)/7.6‰\right)+1\right],$$

where ‐3.4 ‰ is the difference between δ^15^N_GLU_—δ^15^N_PHE_ in primary producers, and 7.6 ‰ is the trophic discrimination factor, representing the amount it takes for an organism to shift from one trophic level to the next.

(4) Trophicposition∼Treatment

## 3 RESULTS

### 3.1 Detecting the magnitude of change in glutamic acid and phenylalanine profiles over the course of fixation and preservation

#### 3.1.1 Yelloweye Rockfish

**FIGURE 1:**
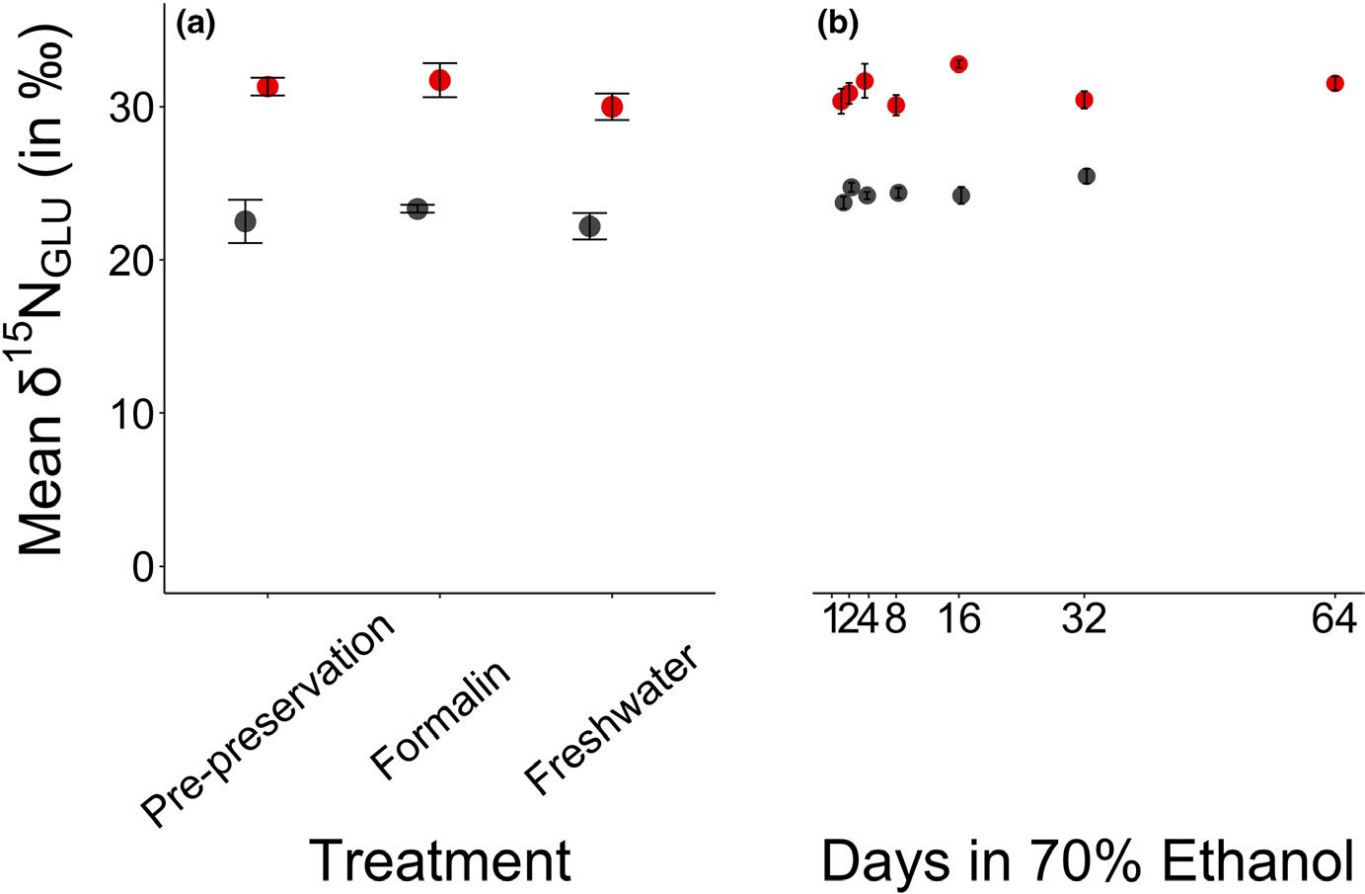
The mean ± 1 *SE* of the δ^15^N_GLU_ (in ‰) for Yelloweye Rockfish (*Sebastes ruberrimus*; in red) and Pacific Herring (*Clupea pallasii*; in gray) of each sample taken. Panel (a) refers to treatment type, and Panel (b) refers to Days in 70% ethanol

**FIGURE 2:**
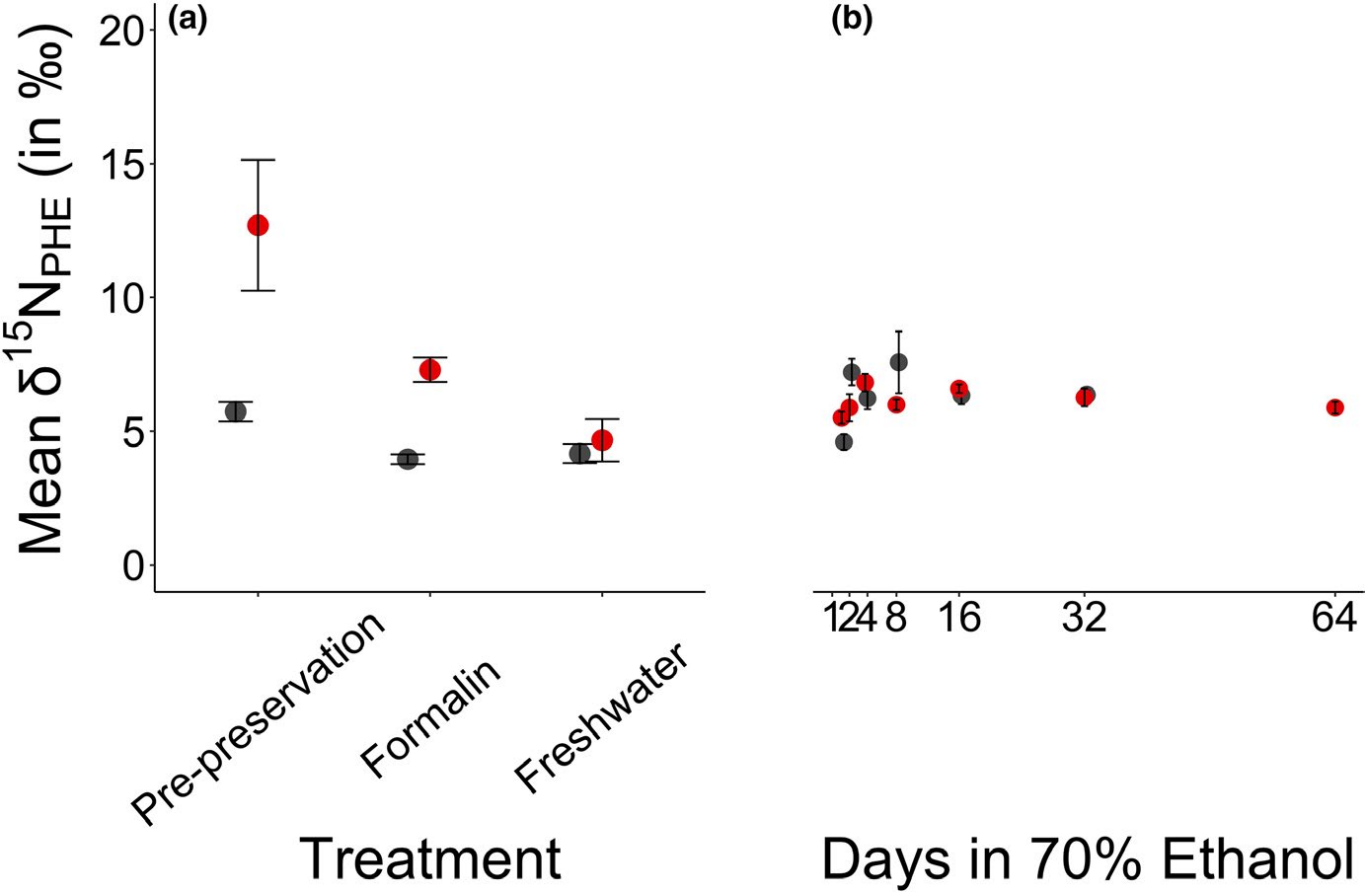
The mean ± 1 *SE* of the δ^15^N_PHE_ (in ‰) for Yelloweye Rockfish (*Sebastes ruberrimus*; in red) and Pacific Herring (*Clupea pallasii*; in gray) of each sample taken. Panel (a) refers to treatment type, and Panel (b) refers to Days in 70% ethanol

Compared to pre‐preservation samples, the δ^15^N_GLU_ and δ^15^N_PHE_ values of day 64 samples were depleted by 0.9 ± 0.6‰ and 6.4 ± 2.2‰, respectively. The δ^15^N_GLU_ values of fish tissue were statistically similar across all sampling points, including the pre‐preservation sample (Table 1, Figure 1, Appendix S6). The δ^15^N_PHE_ values were significantly different among the pre‐preservation, formalin, and freshwater samples, and when comparing the pre‐preservation tissue to tissues preserved for 2, 4, 8, 32, and 64 days in 70% ethanol (Table 1, Figure 2, Appendix S6). None of the pairwise comparisons of tissues preserved in ethanol indicated statistically significant differences within pairs (Table 1, Figure 2, Appendix S6).

**TABLE 1:** Statistically significant pairwise comparisons of the generalized linear models of the source amino acid (Phenylalanine) values of Yelloweye Rockfish (*Sebastes ruberrimus)* including stepwise‐p and FDR‐corrected (*p*‐bh) *p*‐values

Pairwise comparison of treatments and/or days in 70% ethanol	Phenylalanine				
	Est	*SE*	*z*	*p*	*p*‐bh
Pre‐preservation—Formalin	−0.058	0.015	−3.962	**.003**	**.003**
Pre‐preservation—Freshwater	−0.136	0.021	−6.373	**.000**	**.000**
Freshwater—Formalin	0.077	0.024	3.256	**.036**	**.035**
Pre‐preservation—1	−0.103	0.018	−5.577	**.000**	**.000**
Pre‐preservation—2	−0.068	0.016	−4.378	**.001**	**.001**
Pre‐preservation—4	−0.073	0.016	−4.584	**.000**	**.000**
Pre‐preservation—8	−0.091	0.017	−5.230	**.000**	**.000**
Pre‐preservation—16	−0.091	0.020	−4.629	**.000**	**.000**
Pre‐preservation—32	−0.088	0.017	−5.127	**.000**	**.000**
Pre‐preservation—64	−0.081	0.017	−4.878	**.000**	**.000**

Note

Non‐significant comparisons for glutamic acid and phenylalanine are listed in Appendix S6. Bolded values are statistically significant at the *p* = .05 level.

#### 3.1.2 Pacific Herring

Compared to pre‐preservation samples, the δ^15^N_GLU_ and δ^15^N_PHE_ values of day 32 samples were depleted by 1.9 ± 2.5‰ and by 2.2 ± 1.9‰, respectively (Figures 1 and 2). The δ^15^N_GLU_ of fish tissues was statistically similar across all tissues except between pre‐preservation and day 8, and freshwater and day 8, where pre‐preservation tissues were significantly enriched (Table 2, Appendix S6, Figure 1). The δ^15^N_PHE_ values of fish tissues were statistically similar across all samples after day 1 in 70% ethanol, but varied before this time (Table 2, Figure 2, Appendix S6).

**TABLE 2:** Statistically significant pairwise comparisons of the generalized linear models of glutamic acid and phenylalanine values of Pacific Herring (*Clupea pallasii*) including stepwise‐p and FDR‐corrected (*p*‐bh) *p*‐values

Pairwise comparison of treatments and/or days in 70% ethanol	Glutamic acid					Phenylalanine				
	Est	*SE*	*z*	*p*	*p*‐bh	Est	*SE*	*z*	*p*	*p*‐bh
Pre‐preservation—Formalin						−0.025	0.008	−3.285	**.028**	**.027**
Formalin—2						0.030	0.007	4.190	**.001**	**.001**
Formalin—4						0.031	0.007	4.361	**.001**	**.000**
Formalin—8						0.032	0.007	4.408	**.000**	**.000**
Formalin—16						0.038	0.007	5.477	**.000**	**.000**
Formalin—32						0.040	0.007	5.762	**.000**	**.000**
Freshwater—2						0.027	0.007	3.769	**.005**	**.005**
Freshwater—4						0.028	0.007	3.941	**.002**	**.003**
Freshwater—8	−3.273	0.916	−3.573	.011	.011	0.028	0.007	3.988	**.002**	**.002**
Freshwater—16						0.035	0.007	5.064	**.000**	**.000**
Freshwater—32						0.037	0.007	5.352	**.000**	**.000**
16–1						−0.028	0.007	−4.135	**.001**	**.001**
32–1						−0.029	0.007	−4.427	**.000**	**.000**

Note

Non‐significant comparisons for glutamic acid and phenylalanine are included in Appendix S6. Bolded values are statistically significant at the *p* = .05 level.

### 3.2 Assessing when change in glutamic acid and phenylalanine profiles stabilizes

#### 3.2.1 Yelloweye Rockfish

**FIGURE 3:**
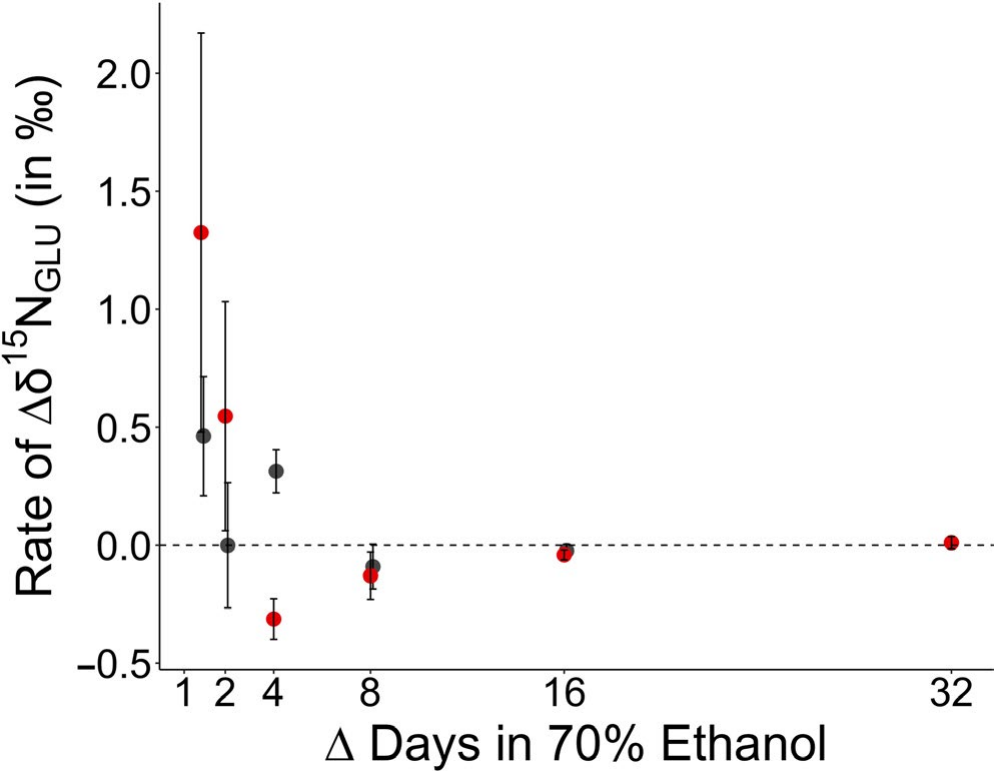
The mean ± 1 *SE* of the rate of change in δ^15^N_GLU_ (in ‰) for Yelloweye Rockfish (*Sebastes ruberrimus*; in red) and Pacific Herring (*Clupea pallasii*; in gray) over the change in time (in days)

**FIGURE 4:**
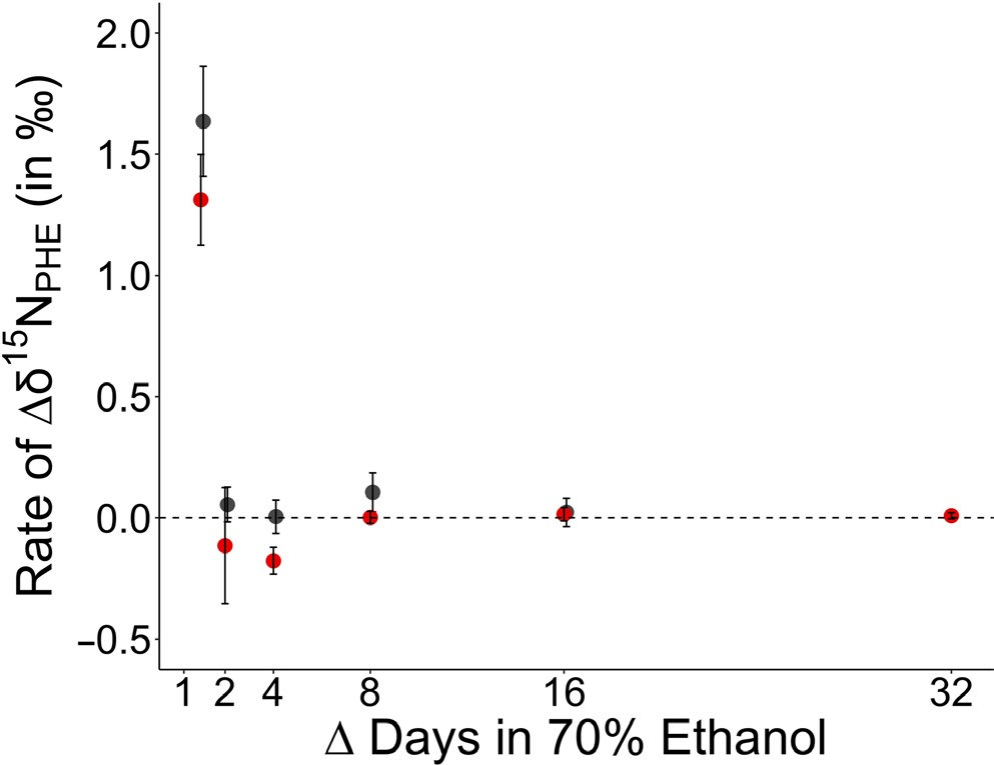
The mean ± 1 *SE* of the rate of change in δ^15^N_PHE_ (in ‰) for Yelloweye Rockfish (*Sebastes ruberrimus*; in red) and Pacific Herring (*Clupea pallasii*; in gray) over the change in time (in days)

The rate of change in the ethanol‐preserved tissue after 32 days for δ^15^N_GLU_ and δ^15^N_PHE_ values was 0.0 ± 0.0‰ and 0.0 ± 0.0‰, respectively (Figures 3, 4). The rate at which change occurred was only statistically different between days 1 and day 2, and days 2 and 4 for δ^15^N_PHE_ values (Table 3, Figures 3, 4). By the last sampling point in the study (64 days), the rate of change reached a slope of zero (Figures 3, 4).

**TABLE 3:** Pairwise comparisons of the generalized linear models of the rate of change of trophic and source amino acid values of Yelloweye Rockfish (*Sebastes ruberrimus*) and Pacific Herring (*Clupea pallasii*) samples in 70% ethanol, including stepwise‐p and FDR‐corrected (*p*‐bh) *p*‐values

Pairwise comparison	Glutamic acid					Phenylalanine				
	Est	*SE*	*z*	*p*	*p*‐bh	Est	*SE*	*z*	*p*	*p*‐bh
**Rockfish**										
2–1	−0.297	0.161	−1.846	.510	.510	0.517	0.094	5.489	**.000**	**.000**
4–2	0.162	0.147	1.108	.924	.924	−0.614	0.094	−6.518	**.000**	**.000**
8–4	0.347	0.196	1.770	.562	.561	0.002	0.094	0.026	1.000	1.000
16–8	0.138	0.228	0.604	.997	.997	−0.095	0.102	−0.934	.967	.967
32–16	−0.054	0.225	−0.240	1.000	1.000	0.007	0.109	0.060	1.000	1.000
64–32	−0.026	0.203	−0.129	1.000	1.000	−0.003	0.102	−0.029	1.000	1.000
**Herring**										
2–1	0.191	0.141	1.355	.754	.754	−0.618	0.068	−9.143	**.000**	**.000**
4–2	−0.283	0.141	−2.009	.337	.337	0.571	0.066	8.694	**.000**	**.000**
8–4	0.236	0.141	1.674	.549	.549	0.047	0.084	0.558	.993	.993
16–8	0.196	0.141	1.390	.733	.733	0.090	0.083	1.089	.881	.881
32–16	0.040	0.141	0.287	1.000	1.000	0.071	0.082	0.861	.953	.953

Note

Bolded values are statistically significant at the *p* = .05 level.

#### 3.2.2 Pacific Herring

The rate of change in the ethanol‐preserved tissue after 16 days for δ^15^N_GLU_ and δ^15^N_PHE_ values was 0.0 ± 0.0‰ and 0.0 ± 0.1‰, respectively. The rate at which change occurred was only statistically different between days 1 and day 2, and days 2 and 4 for δ^15^N_PHE_ values (Table 3, Figures 3, 4). The rate of change approached zero by the last sampling point in the study (32 days, Figures 3, 4).

### 3.3 Detecting changes in estimated trophic position over the course of preservation

#### 3.3.1 Yelloweye Rockfish

**FIGURE 5:**
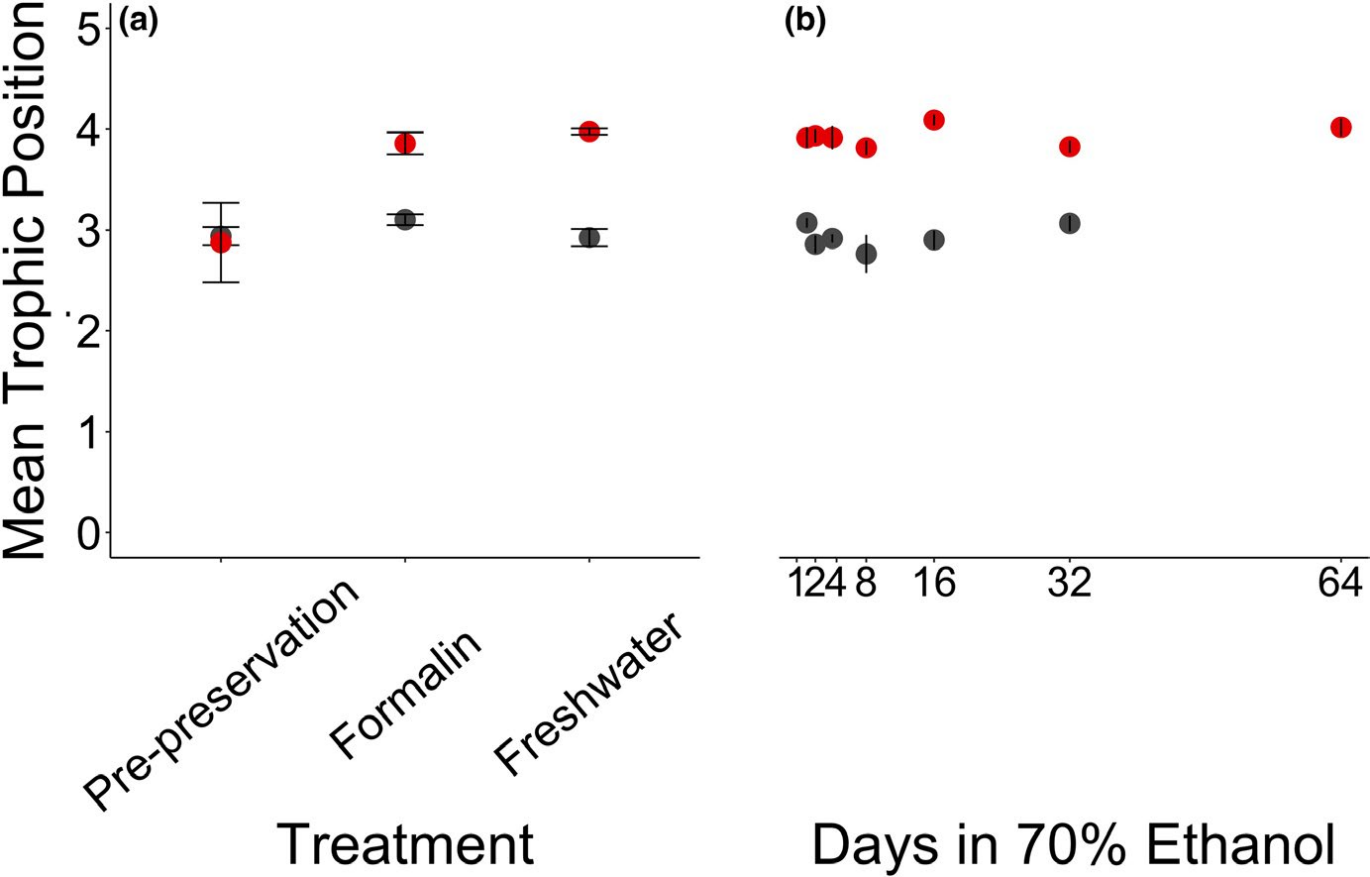
The mean ± 1 *SE* of the estimated trophic position for Yellowfish Rockfish (*Sebastes ruberrimus*; in red) and Pacific Herring (*Clupea pallasii*; in gray) of each sample taken. Panel (a) refers to treatment type and Panel (b) refers to Days in 70% ethanol

On average, the trophic position of the day 64 sample was 0.992 ± 0.357 greater than the initial pre‐preservation sample. The trophic position of the pre‐preservation sample was significantly lower than the trophic position of all other samples, and the trophic positions of all samples preserved in 70% ethanol were statistically similar (Table 4, Figure 5, Appendix S7).

**TABLE 4:** Statistically significant pairwise comparisons of the generalized linear models examining the trophic position of tissue samples of Yelloweye Rockfish (*Sebastes ruberrimus)* across treatments and/or days in 70% ethanol

Pairwise comparison	Est	*SE*	Z	*p*	*p*‐bh
Pre‐preservation—Formalin	−0.982	0.179	−5.499	**.000**	**.000**
Pre‐preservation—Freshwater	−1.100	0.179	−6.157	**.000**	**.000**
Pre‐preservation—1	−1.037	0.179	−5.808	**.000**	**.000**
Pre‐preservation—2	−1.039	0.179	−5.819	**.000**	**.000**
Pre‐preservation—4	−1.213	0.179	−6.795	**.000**	**.000**
Pre‐preservation—8	−1.142	0.179	−6.397	**.000**	**.000**
Pre‐preservation—16	−1.056	0.191	−5.534	**.000**	**.000**
Pre‐preservation—32	−0.938	0.179	−5.255	**.000**	**.000**
Pre‐preservation—64	−0.950	0.179	−5.318	**.000**	**.000**

Note

Non‐significant pairwise comparisons for Yelloweye Rockfish and Pacific Herring (*Clupea pallasii*) are included in Appendix S7. Bolded values are statistically significant at the *p* = .05 level.

#### 3.3.2 Pacific Herring

On average, the trophic position of day 32 sample was 0.177 ± 0.223 less than the initial pre‐preservation samples. The trophic position of all samples was statistically similar (Table 4, Figure 5, Appendix S7).

## 4 DISCUSSION

Overall, the results of our study suggest that the trophic position of fluid‐preserved specimens can be estimated using CSIA‐AA‐N and that CSIA‐AA‐N estimates for fluid‐preserved specimens should be reported as relative differences. These recommendations are a product of our main findings: the δ^15^N_GLU_ and δ^15^N_PHE_ values per individual were inconsistent in direction and magnitude at the earlier stages of the preservation process but stabilized in similar ways among conspecifics by ~32 and 64 days, for Pacific Herring and Yelloweye Rockfish, respectively. The final phenylalanine values for Yelloweye Rockfish amino acid values were significantly different than the initial pre‐preservation samples. Nonetheless, significant differences in δ^15^N_GLU_ and δ^15^N_PHE_ values were not detected among samples that were in 70% ethanol for >24 hr. This suggests that, although absolute estimates of trophic position cannot be obtained from specimens in natural history collections, relative estimates of trophic position are likely to be reliable, and timelines of trophic position change can be developed by comparing specimens collected at different points in time. Given how different fishes may respond to fixation and preservation differently, we suggest that the utility of general cross‐study correction values is not recommended (e.g., Carabel et al., 2009; Kelly et al., 2006).

The considerable depletion we observed in phenylalanine and the lack of consistency in results across the available preservation studies including our own is the reason we suggest trophic position studies should examine relative and not absolute values of trophic position. We observed a significant depletion effect in phenylalanine, and for only one Pacific Herring time point comparison (freshwater and day 8 in 70% ethanol) did we observe a significant effect on glutamic acid. In contrast, Chua et al. (2020) observed no effects of preservation, and Durante et al. (2020) observed significant enrichment effects for phenylalanine and glutamic acid. Hetherington et al. (2019) observed no effects of preservation on glutamic acid, and observed an enrichment effect for phenylalanine, but suggested this enrichment might be related to poor chromatographic separation. Considering that phenylalanine is subtracted from glutamic acid in the trophic position formula (see Methods), declining phenylalanine values with a glutamic acid value that does not change proportionally can inflate trophic position values. Therefore, it will be essential that studies present the relative differences in the trophic position of similarly treated fluid‐preserved specimens to accurately depict historical change in trophic position.

Determining the mechanisms by which changes in δ^15^N_GLU_ and δ^15^N_PHE_ values occur was not an aim of this study, but we suggest that the continuous passive exchange of compounds between the fish and the fixative probably explains the enrichment and depletion effects we observed. In bulk stable isotope studies, formalin and ethanol have been reported to significantly alter bulk stable carbon and nitrogen isotopic readings (e.g., Ruiz‐Cooley et al., 2011; Sarakinos et al., 2002; Sweeting et al., 2004), probably due to protein hydrolysis and lipid degradation. Neither formalin nor ethanol contains nitrogen, so changes in amino acid values may result from fixatives breaking down or altering nitrogenous bonds within the fish. The lack of patterns in these leaching effects may be related to individual variation, because each fish's condition somewhat varies with respect to protein and lipid composition.

The vast number of fluid‐preserved specimens that exist globally provides a strong impetus for trophic ecologists to use CSIA‐AA‐N on specimens held in natural history collections. We suggest that, before CSIA‐AA‐N of fluid‐preserved specimens can proceed, we must carefully assess several parameters that could influence the reliability of estimates derived by CSIA‐AA‐N (Popp et al., 2007). Several previous studies have attempted to assess the reliability of CSIA‐AA‐N estimates, with the greatest differences among these studies being: (1) the type and amount of tissue used for analysis, (2) the fixatives used and the amount of time during which the tissues were subjected to each fixative, (3) the amount of replication within species, and (4) the statistical approaches used. Below, we discuss how each of these considerations can alter conclusions concerning the reliability of CSIA‐AA‐N for use on fluid‐preserved specimens and show how our study partially addresses these concerns.

The most critical aspect of the preservation of fluid‐preserved specimens is the choice of tissue. Tissues can vary in their relative proportion of lipid to protein and mass (e.g., Love, 1957). Whereas the former can potentially influence the fixation process itself, the latter may influence the rate at which fixation occurs (Simmons, 2014). In natural history collections, fish specimens are typically preserved whole, which is why we used whole fish in our experiments. At each time point, we sampled a small piece of muscle tissue from under the dorsal fin and above the lateral line, which is standard procedure in fish isotope studies (e.g., Bowes et al., 2020; Welicky et al., 2017). Of the three available studies that preceded our own, two diverged from this method. Chua et al. (2020) and Hetherington et al. (2019) extracted a 5‐ × 5‐ × 15‐mm and 1‐g muscle tissue samples, respectively, and then subjected these small pieces of tissue to their preservation protocols, such that they did not preserve whole fish. Chua et al. (2020) states that, “The high surface area‐to‐volume ratio of our tissue samples (compared to whole museum specimens) aids in ensuring comparable levels of tissue penetration by preservative fluids”. However, these samples are not directly comparable to the state of fishes held in natural history collections, because the hydrolysis of proteins during fixation should be different for tissues of differing composition, and a pure small muscle sample lacks the majority of lipid and collagen found in a whole fish (i.e., skin, scale, circulatory, digestive, and reproductive organs), such that these samples are unique in composition. Additionally, the leaching of compounds from the preserved material into the fixative solution (and potentially back into the preserved material) may also influence fixation, and this process is probably different for tissues with differing composition as well (Simmons, 2014). For these reasons, we preserved whole fish to mirror the standard preservation process of natural history collections and then we sampled each fish from under the dorsal fin, mirroring the standard sampling process of isotope studies.

The fixative chemical and the time spent in that chemical may also play a significant role in CSIA‐AA‐N‐derived estimates. In natural history collections, whole fish are generally submerged in 10% formalin for 1–2 weeks, depending on size, followed by two consecutive 24 hr freshwater baths, before finally being placed in a fixative of 70% ethanol or graduated up in a fixative from ~40% to 70% ethanol over several weeks (e.g., Simmons, 2014). Hetherington et al. (2019) diverged from these methods by using 95% ethanol as their final fixative. Durante et al. (2020) graduated ethanol from 50% to 70% weekly for 3 weeks. Although ethanol lacks nitrogenous compounds, a stronger concentration of ethanol may increase the rate at which lipids leach from tissues. This may be particularly evident during the initial days/weeks of fixation, and Chua et al. (2020) noted that in bulk studies most isotopic shifts occurred in the first 3 weeks. To our knowledge, our study is the only one that collected data regularly from the onset of ethanol preservation, whereas other studies made first comparisons of non‐fixed samples to formalin‐ethanol samples after 7 weeks (Chua et al., 2020), 3 months (Durante et al., 2020), and 6 months (Hetherington et al., 2019). In order to measure the effect of preservation time on δ^15^N profiles (a recommendation of Chua et al., 2020), we captured changes in the amino acid values over each step of the preservation process and determined the point at which preservation effects stabilize. Similar to bulk studies, we observed the greatest shifts in isotopic composition within the first 2 weeks of our study, and isotopic shifts were statistically negligible after 24 hr in ethanol.

We observed that the isotopic changes in our samples were the most prominent at the formalin fixation stage. Coincidentally, this step is the least repeatable part of the preservation process and has rarely been singled‐out in preservation studies. In fact, our study and that of Durante et al. (2020),   are the only works to provide information about the effects of formalin fixation on a fish sample, independent of any other chemical preservation step. Past bulk stable isotope results have suggested formalin fixation would not have a significant impact on isotopic values (e.g., Lau et al., 2012; Rennie et al., 2012), and a similar finding for CSIA‐AA was reported by Ogawa et al. (2013). However, this study fixed a muscle sample (not whole fish) in 5% formalin and compared this sample to frozen tissue after 62 weeks. From reviewing our findings and that of others, we suggest further investigations into the effects of formalin fixation alone are warranted, and careful consideration to sample type, and proportional volume of fish to formalin should be given.

One of the greatest strengths of the CSIA‐AA approach is that samples sizes are smaller because environmental reference samples are unnecessary (e.g., Bowes & Thorp, 2015, reviewed in Chikaraishi et al., 2009); however, we maintain that, in proof‐of‐concept studies such as this one, a larger sample size and replication are critical to establish the validity of the approach. The sample sizes of the three studies that preceded our own were relatively small; the number of individuals used per fish species examined ranged between one and three. Chua et al. (2020) accounted for small sample size by conducting a Bayesian analysis, which is robust to small sample size. Hetherington et al. (2019) conducted pairwise *t*‐tests across multiple taxa (fish, cephalopod, copepod), and for the fish samples within that analysis, one individual was examined between pre‐preservation and 6 months, and two individuals were examined between pre‐preservation and 2 years. Durante et al. (2020) examined one individual of each of two different species, but increased their sample size by taking 6 samples of the same fish at each time point and their raw data report include values from five samples and their mean. To improve upon the currently published methods, we used 4–5 individuals per species and further strengthened our analyses by taking 9–10 samples per individual across time.

The purpose of this study was to improve on the currently published research, but our study also has limitations. We did observe different enrichment/depletion patterns between our two species in response to fixation, but we are unable to determine whether these differences are related to the species being preserved differently, responding differently to the same fluid‐preservation protocol, and/or having inherently different isotopic compositions. We chose to preserve the Yelloweye Rockfish and Pacific Herring differently to adhere to standard natural history collection protocols (i.e., a ventral incision is made in larger fishes, but not in smaller fishes, to allow for better chemical penetration; larger fishes also typically sit in formalin for 1 week longer than do smaller fishes). Consequently, we could not pool across species to improve sample size; simultaneously, given the low level of replication at the species level (*n* = 2) and the individual level (*n* = 4 or 5), we could not examine species‐ or individual‐level effects. Chua et al. (2020) nested individuals within species and determined that there was a significant effect of species but not fluid preservation influencing amino acid values. In contrast, Durante et al. (2020) documented significant effects of fluid preservation, species, and their interaction on amino acid values. More testing is warranted given the previously described limitations of all these studies, including our own. A remaining question from our analysis is: Might amino acid values have changed at time points after the final sample was taken? Although the only way to answer this question satisfyingly is to perform an experiment over a timeline exceeding 32 days for Pacific Herring and 64 days for Yelloweye Rockfish, our rate‐of‐change analyses suggest that changes had diminished to near zero by the end of our experiment. Our results are in agreement with other preservation studies as they found no differences among their samples when examining time periods beyond 2 months (Durante et al., 2020; Hetherington et al., 2019). The fact that the tissue available to sample (i.e., fish muscle tissue) is finite prevents sampling from being indefinite. Nonetheless, we are continuing to sample the Yelloweye Rockfish reported in this study for later analysis and publication. Our study strictly followed the currently used preservation protocols of natural history collections (e.g., Jenkins et al., 2014), but these practices have evolved over time, and curators have not generally documented their preservation practices. Our work and that of other preservation studies cannot predict the variable effects of these preservation protocol differences. The differences in the aforementioned protocols and ours might be linked to why our success varied in obtaining precise data for all amino acids across all our time points. (Bowes et al., 2020; Thorp & Bowes, 2017). Therefore, for CSIA‐AA studies that leverage fluid‐preserved specimens, it will be critical to work with curators to understand the way in which the samples under study were likely preserved, and to investigate how CSIA‐AA‐N‐generated trophic‐level estimates vary with differences in preservation protocols. The urgency of obtaining information on ecosystems of the past demands that we develop reliable methods for extracting this historical information.

Lastly, we note that the challenges that constrain the aforementioned studies and our own are cost, time, and lack of networking/communication among researchers and curators interested in this line of research. With no automation of the wet lab protocols and little automation for data analysis, CSIA‐AA‐N can be prohibitively expensive and time‐consuming. For these reasons, we aimed to improve upon the currently available literature by producing the most well‐replicated dataset of its kind to date and using the most commonly employed preservation method of natural history collections. Our results suggest that CSIA‐AA‐N of fluid‐preserved specimens is appropriate for making relative, within‐species comparisons of estimated trophic level.

## CONFLICT OF INTERESTS

The authors declare there are no competing interests or conflicts of interest.

## AUTHOR CONTRIBUTION

**Rachel L. Welicky:** Conceptualization (lead); Data curation (lead); Formal analysis (lead); Funding acquisition (lead); Investigation (lead); Methodology (lead); Project administration (lead); Resources (equal); Software (equal); Supervision (lead); Validation (lead); Visualization (lead); Writing‐original draft (lead); Writing‐review & editing (lead). **Terry Rolfe:** Data curation (supporting); Methodology (supporting); Writing‐review & editing (supporting). **Karrin Leazer:** Data curation (supporting); Methodology (supporting); Writing‐review & editing (supporting). **Katherine Maslenikov:** Conceptualization (supporting); Methodology (supporting); Writing‐review & editing (supporting). **Luke Tornabene:** Conceptualization (supporting); Methodology (supporting); Writing‐review & editing (supporting). **Gordon Holtgrieve:** Conceptualization (supporting); Formal analysis (supporting); Writing‐review & editing (supporting). **Chelsea Wood:** Conceptualization (supporting); Formal analysis (supporting); Writing‐review & editing (supporting).

### Open Research Badges

This article has been awarded Open Data and Open Materials Badges. All materials and data are publicly accessible via the Open Science Framework at https://doi.org/10.5061/dryad.fn2z34tsc.

## Supporting information

Appendix S1‐S7

## Data Availability

All data and associated R Scripts are accessible at: https://doi.org/10.5061/dryad.fn2z34tsc
