# Supplementary material for: Fluid‐preserved fishes are one solution for assessing historical change in fish trophic level
Source: Ecol Evol. 2020 Nov 25;11(1):415–26. doi: 10.1002/ece3.7061 (PMC7790622; doi:10.1002/ece3.7061)
Supplement: Supplementary file 1 — Appendix S1‐S7 [file ECE3-11-415-s001.docx]

**APPENDIX 1**

*Amino acid derivatization*

We extracted and derivatized amino acids from each fish tissue sample using a modified acetyl chloride-pivaloyl chloride derivatization process based on Metges et al. (1996), Popp et al. (2007), and Chikaraishi et al. (2009). Briefly, 5–6mg of dried tissue was combined with 20 μL of norleucine (0.05 M in 0.1 N HCl), the internal quantification and isotopic standard, and 6N protein-sequencing grade HCl. This mixture was flushed with N_2_, hydrolyzed at 150°C for 70 min, cooled to room temperature, evaporated under N_2_ at 55°C, and then 1 mL of 0.01 N HCl was added to the sample. The samples sat in 0.01 N HCl for 30 minutes with periodic vortexing. Samples were then filtered using a 0.22-μm Millex-GP, and purified using a 5-cm cation-exchange column. The eluent was evaporated to dryness under N_2_ at 80°C, and then 1 mL 0.01 N HCl was added. Following full dissolution after 30 minutes, samples were de-fatted by mixing 2 mL 3:2 n-hexanes/DCM with the sample, followed by centrifuging and removing the upper organic solvent layer three times. To ensure that no organic solvent remained, samples were briefly subjected to N_2_. After this, samples were frozen at -80°C overnight and then freeze dried for ~12–24hr. Next, samples were esterified using 2 mL 4:1 isopropanol:acetyl chloride, were briefly flushed with N_2_, and were then placed in 110°C heat for 2h. Samples were then brought to room temperature and were evaporated at 55°C under N_2._ To derivatize samples, 1 mL of 4:1 toluene:pivaloyl chloride was added to each sample, exposed to N_2_ briefly, and acetylated at 110**°**C for 2h. Samples were brought to room temperature and then were evaporated at room temperature under N_2._ A liquid-liquid extraction was performed by re-dissolving samples in 1mL P buffer water followed by 2mL of 3:2 n-hexanes/DCM, shaking the samples, centrifuging them, and then saving the upper organic fraction in a new vial. Another 1.8mL of 3:2 n-hexanes/DCM was added to each original sample vial and the process was repeated until 3.8mL of n-hexanes/DCM were in the new vial. Next, the n-hexanes/DCM were evaporated at room temperature under N_2_, re-dissolved in 100 μL dried DCM, evaporated at room temperature under N_2_, and raised in 100 μL ethyl acetate. Finally, samples were stored frozen until analysis. A standard amino acid mixture (including glutamic acid and phenylalanine) was also prepared following the above esterification, derivatization, and extraction method.

Individual amino acids were purchased from Sigma Aldrich in powder form. The amino acids included in the standard mixture were alanine, proline, valine, threonine, leucine, norleucine, aspartic acid, glutamic acid, phenylalanine, tyrosine, isoleucine, proline, and serine. Each amino acid was weighed to a target mass achieving 0.05 molarity in 25mL of HCl, except for tyrosine, which is mixed to a 0.0125 molarity in 25mL as a mechanism to avoid dissolution.

**APPENDIX 2**

| Table 1. GC oven temperature ramp program for a single injection. Flow rate was 2ml/min. | | |
| --- | --- | --- |
| Initial Temperature (˚C) | 40 |  |
| Initial Hold (min) | 2.0 |  |
| Ramp 1 (˚C min^-1^) | 8.0 |  |
| Temperature 1 (˚C) | 100 |  |
| Hold 1 (min) | 0.0 |  |
| Ramp 2 (˚C min^-1^) | 6.0 |  |
| Temperature 2 (˚C) | 185 |  |
| Hold 2 (min) | 8.0 |  |
| Ramp 3 (˚C min^-1^) | 9.0 |  |
| Temperature 3 (˚C) | 300 |  |
| Hold 3 (min) | 6.0 |  |

**APPENDIX 3**

*Drift correction of raw data*

Step 1: To quality check and determine the drift across standards, we first calculated the following for each run’s standard injections per amino acid:

Average Retention Time:$\frac{\Sigma Retention time}{number of injections}$

Retention Time Drift: Last injection retention time - First injection retention time

Average Peak Area: $\frac{\Sigma Peak area}{Number of injections}$

Average δ^15^N: $\frac{\Sigma\delta15N}{Number of injections}$

Standard deviation of δ^15^N: $\frac{Average \delta15N}{\sqrt{Number of injections}-1}$

δ^15^N slope: the linear slope of injections 3–8

δ^15^N intercept: the intercept of the linear slope of injections 3–8

Step 2: To quality check and drift-adjust each amino acid for each triplicate sample, we calculated:

Average Retention Time: $\frac{\Sigma Retention time}{Number of injections}$

Average Peak Area: $\frac{\Sigma Peak area}{Number of injections}$

Standard deviation of peak area: $\frac{Average peak area}{\sqrt{Number of injections}-1}$

Average δ^15^N: $\frac{\Sigma\delta15N}{Number of injections}$

Standard deviation of δ^15^N: $\frac{Average \delta15N}{\sqrt{Number of injections}-1}$

δ^15^Ndrift: δ^15^N slope of the respective amino acid x [average analysis number of the specific amino acid - (first analysis number -1)]

δ^15^N drift adjusted: δ^15^N average - δ^15^N drift

Step 3: For each δ^15^N drift adjusted value, a final correction to account for differences between the known AA standard values and the measured standard values was calculated and then applied as follows:

AA Standard difference: Measured average δ^15^N - known per mil δ^15^N

Corrected δ^15^N: δ^15^N drift adjusted - AA Standard difference

**APPENDIX 4**

| Appendix 4. Precision (the standard deviation of the mean) of each amino acid standard (in ‰). | | | |
| --- | --- | --- | --- |
| Amino Acid | Mean | Median | Maximum |
| Alanine | 0.23 | 0.17 | 0.51 |
| Valine | 0.42 | 0.34 | 0.80 |
| Isoleucine | 0.20 | 0.19 | 0.28 |
| Norleucine | 0.47 | 0.35 | 0.95 |
| Proline | 0.51 | 0.47 | 0.78 |
| Threonine | 0.31 | 0.33 | 0.50 |
| Aspartic Acid | 0.36 | 0.30 | 0.66 |
| Serine | 0.38 | 0.38 | 0.48 |
| Glutamic Acid | 0.21 | 0.18 | 0.31 |
| Phenylalanine | 0.33 | 0.31 | 0.51 |
| Tyrosine | 0.32 | 0.35 | 0.41 |

**APPENDIX 5**

| Appendix 5 Table 1. The mean of the δ^15^N (in ‰) for Yelloweye Rockfish (*Sebastes ruberrimus*). ALA = Alanine, VAL= Valine, ISO = Isoleucine, PRO = Proline, THR = Threonine, ASP = Aspartic Acid, SER = Serine, GLU = Glutamic Acid, PHE = Phenylalanine, TYR = Tyrosine. Empty cell means datapoint is unavailable. | | | | | | | | | | | |
| --- | --- | --- | --- | --- | --- | --- | --- | --- | --- | --- | --- |
| Fish Identifier | Treatment | ALA | VAL | ISO | PRO | THR | ASP | SER | GLU | PHE | TYR |
| Rockfish 1 | Pre-preservation | 31.3 | 32.8 | 32.4 | 26.2 | -18.8 |  |  | 33.0 | 10.3 | 10.0 |
|  | Formalin |  |  |  |  |  |  |  | 31.8 | 6.5 |  |
|  | Freshwater |  |  |  |  |  |  |  | 31.5 | 5.5 |  |
|  | 1 | 32.6 | 29.3 | 32.1 |  | -27.2 | 20.8 | 15.2 | 32.6 | 6.0 |  |
|  | 2 | 34.7 | 31.8 | 31.6 | 33.9 | -22.5 |  |  | 34.7 | 7.7 |  |
|  | 4 | 34.0 | 34.9 | 32.3 |  |  |  |  | 33.0 | 6.2 |  |
|  | 8 | 32.5 | 35.6 | 30.6 | 27.3 | -22.2 | 22 | 14.1 | 32.6 | 5.4 |  |
|  | 16 | 31.4 | 33.2 | 31.4 | 28.1 | -22.7 | 24.6 | 12.3 | 31.0 | 5.3 |  |
|  | 32 | 31.7 | 33.6 | 31.3 | 34.5 | -22.0 | 26.8 | 16.0 | 30.9 | 6.3 |  |
|  | 64 | 32.1 | 34.2 | 31.4 | 31.4 | -19.1 | 23.2 | 12.6 | 31.2 | 6 |  |
| Rockfish 2 | Pre-preservation | 29.8 | 34.8 | 31.1 |  | -18.0 |  |  | 31.1 | 15.8 | 9.8 |
|  | Formalin |  |  |  |  |  |  |  | 29.9 | 7.2 |  |
|  | Freshwater |  |  |  |  |  |  |  | 30.9 | 5.9 |  |
|  | 1 | 30.9 | 29.5 | 30.1 | 33.1 | -22.5 | 24.8 | 24.4 | 30.0 | 4.9 |  |
|  | 2 | 31.4 | 33 | 5.2 |  | -20.8 | 23.8 | 22.6 | 30.6 | 6.3 |  |
|  | 4 | 31.4 | 31.8 | 29.8 | 34.0 | -21.4 | 22.0 | 19.1 | 32.8 | 6.9 |  |
|  | 8 | 31.5 | 34.4 | 29.0 | 28.4 | -20.0 | 24.0 | 17.9 | 31.7 | 5.7 |  |
|  | 16 | 30.3 | 32.8 |  | 31.5 | -22.8 | 25.5 | 13.9 | 29.6 | 5.5 |  |
|  | 32 | 29.9 | 32.2 | 28.8 | 30.1 | -19.0 | 23.2 | 11.4 | 28.4 | 5.7 |  |
|  | 64 | 31.0 | 33.2 | 31.6 | 35.3 | -19.7 | 23.7 | 13.8 | 31.1 | 7.2 |  |
| Rockfish 3 | Pre-preservation | 30.4 | 31.5 | 29.6 | 24.6 | -21.3 | 24.0 | 13.2 | 30.6 | 7.1 | 12.8 |
|  | Formalin | 28.5 | 32.5 | 29.8 | 27.7 | -17.6 |  |  | 30.3 | 6.9 |  |
|  | Freshwater |  |  |  |  |  |  |  | 30.0 | 4.9 |  |
|  | 1 | 31.1 | 28 | 29.4 |  | -20.8 | 22.9 | 19.5 | 30.2 | 5.6 |  |
|  | 2 | 29.8 | 29.7 | 30.0 |  | -22 | 22.4 | 16.3 | 29.6 | 6.5 |  |
|  | 4 | 31.6 | 33.7 | 29.3 | 31.9 | -20.7 | 20.4 | 15.4 | 32.1 | 6.8 |  |
|  | 8 | 30.2 | 31.3 | 28.7 | 18.1 | -18.8 | 19.9 | 14.1 | 30.4 | 5.9 |  |
|  | 16 | 30.0 | 32.3 | 29.0 | 30.1 | -21.4 | 22.0 | 11.3 |  |  |  |
|  | 32 | 30.7 | 33.5 | 30.2 | 28.3 | -19.8 | 23.9 | 14.0 | 29.6 | 5.6 |  |
|  | 64 | 29.9 | 31.9 | 28.8 | 28.9 | -16.3 | 21.2 | 13.1 | 28.8 | 5.6 |  |
| Rockfish 4 | Pre-preservation | 28.5 | 28.1 | 30.4 |  | -19.5 |  |  | 30.5 | 17.6 |  |
|  | Formalin | 31.7 | 32.8 | 30.2 | 30.3 | -19.4 |  |  | 34.8 | 8.6 |  |
|  | Freshwater |  |  |  |  |  |  |  | 27.6 | 2.4 |  |
|  | 1 | 29.8 | 30.5 | 31.7 |  | -24.9 | 23.1 | 27.3 | 28.6 | 5.5 |  |
|  | 2 | 30.5 | 30.0 | 31.7 |  | -23.8 | 20.7 | 16.5 | 31.8 | 6.8 |  |
|  | 4 | 33.8 | 31.2 | 30.6 | 31.5 | -21.2 | 20.8 | 15.2 | 33.3 | 6.6 |  |
|  | 8 | 31.5 | 33.2 | 30.7 |  | -22.9 | 21 | 14.6 | 31.4 | 6.5 |  |
|  | 16 | 31.1 | 32.3 | 30.6 | 29.8 | -22.5 | 21.8 | 12.9 | 32 | 6.9 |  |
|  | 32 | 30.6 | 33.5 | 29.6 | 28.2 | -22.0 | 20.7 | 14.0 | 31.3 | 6.4 |  |
|  | 64 | 31.6 | 33.0 | 26.6 | 23.3 | -29.5 | 21.9 | 8.9 | 30.6 | 6.3 |  |

| Appendix 5 Table 2. The mean of the δ^15^N (in ‰) for Pacific Herring (*Clupea pallassi*). ALA = Alanine, VAL= Valine, ISO = Isoleucine, PRO = Proline, THR = Threonine, ASP = Aspartic Acid, SER = Serine, GLU = Glutamic Acid, PHE = Phenylalanine, TYR = Tyrosine. Empty cell means datapoint is unavailable. | | | | | | | | | | | |
| --- | --- | --- | --- | --- | --- | --- | --- | --- | --- | --- | --- |
| Fish Identifier | Treatment | ALA | VAL | ISO | PRO | THR | ASP | SER | GLU | PHE | TYR |
| Herring 1 | Pre-preservation | 23.0 | 22.8 | 21.9 |  | -6.0 | 18.3 | 12.4 | 22.4 | 6.1 | 10.5 |
|  | Formalin | 21.5 | 22.4 | 18.8 |  | -5.4 | 16.5 | 12.7 | 22.4 | 4.4 |  |
|  | Freshwater | 19.1 | 20.7 |  |  | -7.8 | 13.9 | 9.7 | 18.8 | 3.3 |  |
|  | 1 | 22.5 | 22.5 | 21.7 | 14.5 | -7.5 | 16.6 | 9.6 | 24.1 | 5.3 | 17.2 |
|  | 2 | 25.4 | 23.8 | 21 | 22.8 | -6.2 | 17.7 | 10.1 | 24.6 | 7.1 |  |
|  | 4 | 25.0 | 24.8 | 22.7 | 22.4 | -6.0 | 17.9 | 12.7 | 22.6 | 7.1 |  |
|  | 8 | 25.8 | 25.0 | 23.4 | 24.8 | -6.7 | 19.1 | 11.8 | 25.2 | 6.3 |  |
|  | 16 | 26.2 | 25.2 | 21.6 | 24.4 | -7.3 | 19.2 | 10.6 | 25.3 | 7.3 | 2.1 |
|  | 32 | 25.6 | 27.7 | 24.7 | 26.5 | -7.8 | 16.3 | 14.6 | 24.5 | 5.8 |  |
| Herring 2 | Pre-preservation | 24.6 | 24.5 | 23.1 |  | -7.7 | 18.1 | 13.0 | 24.3 | 5.9 | 13.7 |
|  | Formalin | 23.7 | 21.7 | 17.9 | 20.2 | -7.8 | 16.6 | 11.7 | 23.9 | 4.1 |  |
|  | Freshwater | 24.1 | 24.8 | 20.9 | 26.0 | -7.5 | 17.7 | 12.2 | 23.6 | 5.1 | -1.9 |
|  | 1 | 24.8 | 24.6 | 23.2 | 23.3 | -7.2 |  |  | 25.1 | 4.8 |  |
|  | 2 | 26.2 | 25.5 | 23.6 | 30.6 | -5.9 | 17.8 | 11.8 | 24.9 | 7.3 |  |
|  | 4 | 26.4 | 26.8 | 23.8 | 24.1 | -7.3 | 17.1 | 14.3 | 26.0 | 6.9 | 1.6 |
|  | 8 | 27.5 | 26.9 | 20.9 | 24.7 | -7.6 | 20.0 | 11.5 | 27.4 | 6.5 |  |
|  | 16 | 23.9 | 26.9 | 23.2 | 17.7 | -8.1 | 17.9 | 12.1 | 23.8 | 6.4 |  |
|  | 32 | 25.8 | 28.4 | 26.2 | 20.2 | -7.8 | 15.8 | 16.9 | 25.0 | 5.4 |  |
| Herring 3 | Pre-preservation | 23.7 | 24.7 | 21.4 | 22.9 | -4.1 | 22.2 | 10.8 | 24.3 | 6.3 | 12.3 |
|  | Formalin | 22.2 | 22.8 | 19.8 | 18.7 | -5.1 | 16.0 | 12.5 | 23.1 | 3.8 |  |
|  | Freshwater | 18.7 | 23.0 | 19.7 | 18.6 | -8.1 | 16.8 | 17.5 | 22.8 | 3.4 |  |
|  | 1 | 22.7 | 22.5 | 21.4 | 17.1 | -5.1 |  | 18.3 | 23.1 | 3.8 |  |
|  | 2 | 25.0 | 24.7 | 22.8 |  | -5.3 | 16.0 | 13.5 | 23.8 | 5.4 |  |
|  | 4 | 24.1 | 24.0 | 21.5 | 27.4 | -6.4 | 14.4 | 14.5 | 24.2 | 5.8 | 1.2 |
|  | 8 | 25.3 | 24.7 | 23.4 | 18.2 | -4.6 | 17.3 | 14.2 | 25 | 6.0 |  |
|  | 16 | 26.0 | 25.1 | 21.6 | 10.5 | -5.2 | 18.7 | 25.3 | 24.5 | 8.9 |  |
|  | 32 | 22.7 | 20.0 | 20.4 | 15.7 | -7.9 | 17.9 | 7.3 | 23.2 | 9.3 | 11.6 |
| Herring 4 | Pre-preservation | 24.3 | 24.0 | 21.2 | 20.1 | -8.4 | 17.2 | 10.7 | 24.4 | 4.7 | 10.0 |
|  | Formalin | 23.6 | 23.1 | 19.2 | 19.8 | -7.7 | 16.1 | 10.6 | 23.6 | 4.1 |  |
|  | Freshwater | 22.7 | 22.6 | 21.9 | 17.6 | -6.9 | 15.4 | 12.8 | 22.6 | 4.3 |  |
|  | 1 | 22.0 | 22.3 | 21.9 | 19.2 |  | 12.7 | 15.7 | 22.8 | 4.0 |  |
|  | 2 | 25.7 | 22.4 | 20.2 | 22.9 | -8.2 | 15.2 | 12.9 | 24.0 | 5.3 |  |
|  | 4 | 23.7 | 24.5 | 21.1 | 25.3 | -8.0 | 13.7 | 16.4 | 24.0 | 5.4 | 2.6 |
|  | 8 | 25.9 | 25.4 | 23.9 | 26.4 | -6.6 | 16.8 | 16.0 | 25.0 | 6.1 |  |
|  | 16 | 26.4 | 26.2 | 23.6 | 22.1 | -5.8 | 17.5 | 13.7 | 24.8 | 7.4 |  |
|  | 32 | 24.0 | 27.8 | 25.6 | 22.5 | -5.5 | 11.0 | 17.4 | 24.3 | 11.3 |  |
| Herring 5 | Pre-preservation | 16.5 | 17.0 | 14.0 |  | -12.0 | 13.4 | 10.3 | 17.0 |  | 6.5 |
|  | Formalin | 22.9 | 23.9 | 21.3 | 17.7 | -5.6 |  | 19.5 | 23.7 | 3.3 |  |
|  | Freshwater | 22.2 | 23.2 | 23.5 | 20.2 | -4.9 | 15.8 | 12.3 | 23.1 | 4.8 | 10.7 |
|  | 1 | 22.6 | 24 | 22.4 | 21.8 | -7.1 | 13.0 | 14.3 | 23.7 | 5.0 |  |
|  | 2 | 23.1 | 22.4 | 24.6 | 21 | -7.7 | 13.6 | 15.5 | 23.7 | 6.1 |  |
|  | 4 | 23.0 | 24.4 | 19.0 | 17.2 | -5.7 | 21.1 | 13.0 | 24.2 | 6.5 |  |
|  | 8 | 23.4 | 25.1 | 22.1 |  | -5.8 | 20.6 | 8.9 | 24.7 | 6.9 |  |
|  | 16 | 26.5 | 28.4 | 25.8 | 25.4 | -7.8 | 18.3 | 11.6 | 25.3 | 6.0 |  |
|  | 32 | 23.8 | 25.8 | 24.7 | 20.0 | -6.0 | 16.3 | 14.3 | 24.8 | 6.2 |  |
|  |  |  |  |  |  |  |  |  |  |  |  |

| **APPENDIX 6** | | | | | | | | | | | |
| --- | --- | --- | --- | --- | --- | --- | --- | --- | --- | --- | --- |
|  | | | | | | | | | | | |
| Appendix 6 Table 1. All pairwise comparisons of the generalized linear models of the trophic and source amino acid values for Yelloweye Rockfish (*Sebastes ruberrimus)* including stepwise-p and FDR-corrected (p-bh) p-values. | | | | | | | | | | | |
|  | Glutamic Acid | | | | | Phenylalanine | | | | |  |
| Pairwise comparison | est | se | z | p | p-bh | est | se | z | p | p-bh |  |
| Pre-preservation - Formalin | -0.411 | 1.066 | -0.385 | 1.000 | 1.000 | -0.058 | 0.015 | -3.962 | **0.003** | **0.003** |  |
| Pre-preservation - Freshwater | 1.322 | 1.066 | 1.240 | 0.966 | 0.966 | -0.136 | 0.021 | -6.373 | **0.000** | **0.000** |  |
| Pre-preservation - 1 | 0.955 | 1.066 | 0.896 | 0.997 | 0.997 | -0.103 | 0.018 | -5.577 | **0.000** | **0.000** |  |
| Pre-preservation - 2 | -0.371 | 1.066 | -0.348 | 1.000 | 1.000 | -0.068 | 0.016 | -4.378 | **0.001** | **0.001** |  |
| Pre-preservation - 4 | -1.466 | 1.066 | -1.375 | 0.935 | 0.935 | -0.073 | 0.016 | -4.584 | **0.000** | **0.000** |  |
| Pre-preservation - 8 | -0.217 | 1.066 | -0.204 | 1.000 | 1.000 | -0.091 | 0.017 | -5.230 | **0.000** | **0.000** |  |
| Pre-preservation - 16 | 0.431 | 1.152 | 0.375 | 1.000 | 1.000 | -0.091 | 0.020 | -4.629 | **0.000** | **0.000** |  |
| Pre-preservation - 32 | 1.221 | 1.066 | 1.146 | 0.980 | 0.980 | -0.088 | 0.017 | -5.127 | **0.000** | **0.000** |  |
| Pre-preservation - 64 | 0.863 | 1.066 | 0.809 | 0.998 | 0.998 | -0.081 | 0.017 | -4.878 | **0.000** | **0.000** |  |
| Freshwater - Formalin | -1.732 | 1.066 | -1.625 | 0.836 | 0.836 | 0.077 | 0.024 | 3.256 | **0.036** | **0.035** |  |
| Formalin - 1 | 1.366 | 1.066 | 1.281 | 0.958 | 0.958 | -0.044 | 0.021 | -2.098 | 0.514 | 0.514 |  |
| Formalin - 2 | 0.040 | 1.066 | 0.037 | 1.000 | 1.000 | -0.010 | 0.019 | -0.509 | 1.000 | 1.000 |  |
| Formalin - 4 | -1.055 | 1.066 | -0.990 | 0.993 | 0.993 | -0.015 | 0.019 | -0.769 | 0.999 | 0.999 |  |
| Formalin - 8 | 0.193 | 1.066 | 0.181 | 1.000 | 1.000 | -0.033 | 0.020 | -1.618 | 0.830 | 0.830 |  |
| Formalin - 16 | 0.842 | 1.152 | 0.731 | 0.999 | 0.999 | -0.033 | 0.022 | -1.471 | 0.897 | 0.897 |  |
| Formalin - 32 | 1.632 | 1.066 | 1.531 | 0.880 | 0.880 | -0.030 | 0.020 | -1.480 | 0.894 | 0.894 |  |
| Formalin - 64 | 1.273 | 1.066 | 1.194 | 0.973 | 0.973 | -0.023 | 0.020 | -1.148 | 0.978 | 0.978 |  |
| Freshwater - 1 | -0.367 | 1.066 | -0.344 | 1.000 | 1.000 | 0.033 | 0.026 | 1.250 | 0.961 | 0.961 |  |
| Freshwater - 2 | -1.693 | 1.066 | -1.588 | 0.854 | 0.854 | 0.068 | 0.024 | 2.797 | 0.131 | 0.130 |  |
| Freshwater - 4 | -2.788 | 1.066 | -2.615 | 0.210 | 0.211 | 0.063 | 0.024 | 2.556 | 0.228 | 0.229 |  |
| Freshwater - 8 | -1.539 | 1.066 | -1.444 | 0.913 | 0.913 | 0.044 | 0.025 | 1.736 | 0.763 | 0.763 |  |
| Freshwater - 16 | -0.890 | 1.152 | -0.773 | 0.999 | 0.999 | 0.044 | 0.027 | 1.639 | 0.819 | 0.819 |  |
| Freshwater - 32 | -0.100 | 1.066 | -0.094 | 1.000 | 1.000 | 0.047 | 0.025 | 1.873 | 0.673 | 0.674 |  |
| Freshwater - 64 | -0.459 | 1.066 | -0.431 | 1.000 | 1.000 | 0.055 | 0.025 | 2.196 | 0.444 | 0.445 |  |
| 2-1 | 1.326 | 1.066 | 1.244 | 0.965 | 0.965 | -0.035 | 0.022 | -1.607 | 0.836 | 0.836 |  |
| 4-1 | 2.421 | 1.066 | 2.271 | 0.408 | 0.408 | -0.030 | 0.022 | -1.352 | 0.937 | 0.936 |  |
| 8-1 | 1.172 | 1.066 | 1.100 | 0.985 | 0.985 | -0.012 | 0.023 | -0.498 | 1.000 | 1.000 |  |
| 16 - 1 | 0.524 | 1.152 | 0.455 | 1.000 | 1.000 | -0.012 | 0.025 | -0.468 | 1.000 | 1.000 |  |
| 32 - 1 | -0.266 | 1.066 | -0.250 | 1.000 | 1.000 | -0.015 | 0.023 | -0.639 | 1.000 | 1.000 |  |
| 64 - 1 | 0.092 | 1.066 | 0.087 | 1.000 | 1.000 | -0.022 | 0.023 | -0.975 | 0.993 | 0.993 |  |
| 4-2 | 1.095 | 1.066 | 1.027 | 0.991 | 0.991 | 0.005 | 0.020 | 0.261 | 1.000 | 1.000 |  |
| 8-2 | -0.154 | 1.066 | -0.144 | 1.000 | 1.000 | 0.023 | 0.021 | 1.119 | 0.981 | 0.981 |  |
| 32 - 2 | -1.592 | 1.066 | -1.494 | 0.895 | 0.895 | 0.020 | 0.021 | 0.978 | 0.993 | 0.993 |  |
| 64 - 2 | -1.234 | 1.066 | -1.157 | 0.978 | 0.978 | 0.013 | 0.020 | 0.643 | 1.000 | 1.000 |  |
| 8-4 | -1.249 | 1.066 | -1.171 | 0.977 | 0.977 | 0.018 | 0.021 | 0.861 | 0.997 | 0.997 |  |
| 64 - 4 | -2.328 | 1.066 | -2.184 | 0.467 | 0.468 | 0.008 | 0.021 | 0.383 | 1.000 | 1.000 |  |
| 2-16 | 0.803 | 1.152 | 0.697 | 1.000 | 1.000 | -0.023 | 0.023 | -1.021 | 0.990 | 0.990 |  |
| 4-16 | 1.897 | 1.152 | 1.648 | 0.824 | 0.824 | -0.018 | 0.023 | -0.786 | 0.999 | 0.999 |  |
| 8-16 | 0.649 | 1.152 | 0.563 | 1.000 | 1.000 | 0.000 | 0.024 | 0.005 | 1.000 | 1.000 |  |
| 32 - 16 | -0.790 | 1.152 | -0.686 | 1.000 | 1.000 | -0.003 | 0.024 | -0.127 | 1.000 | 1.000 |  |
| 64 - 16 | -0.431 | 1.152 | -0.374 | 1.000 | 1.000 | -0.010 | 0.024 | -0.437 | 1.000 | 1.000 |  |
| 4-32 | 2.687 | 1.066 | 2.521 | 0.257 | 0.256 | -0.015 | 0.021 | -0.720 | 0.999 | 0.999 |  |
| 8-32 | 1.439 | 1.066 | 1.349 | 0.942 | 0.942 | 0.003 | 0.022 | 0.142 | 1.000 | 1.000 |  |
| 64 - 32 | 0.359 | 1.066 | 0.336 | 1.000 | 1.000 | -0.007 | 0.022 | -0.337 | 1.000 | 1.000 |  |
| 8-64 | 1.080 | 1.066 | 1.013 | 0.992 | 0.992 | 0.010 | 0.022 | 0.480 | 1.000 | 1.000 |  |

| Appendix 6 Table 2. Pairwise comparisons of the generalized linear models of the trophic and source amino acid values for Pacific Herring (*Clupea pallasii*) including stepwise-p and FDR-corrected (p-bh) p-values. | | | | | | | | | | |
| --- | --- | --- | --- | --- | --- | --- | --- | --- | --- | --- |
|  | Glutamic acid | | | | | Phenylalanine | | | | |
| Pairwise comparison | est | se | z | p | p-bh | est | se | z | p | p-bh |
| Pre-preservation - Formalin | -0.827 | 0.916 | -0.902 | 0.993 | 0.993 | -0.025 | 0.008 | -3.285 | **0.028** | **0.027** |
| Pre-preservation - Freshwater | 0.320 | 0.916 | 0.350 | 1.000 | 1.000 | -0.022 | 0.008 | -2.877 | 0.093 | 0.093 |
| Pre-preservation - 1 | -1.233 | 0.916 | -1.346 | 0.918 | 0.917 | -0.014 | 0.007 | -1.964 | 0.565 | 0.566 |
| Pre-preservation - 2 | -1.696 | 0.916 | -1.851 | 0.648 | 0.647 | 0.005 | 0.007 | 0.715 | 0.999 | 0.999 |
| Pre-preservation - 4 | -1.696 | 0.916 | -1.851 | 0.648 | 0.647 | 0.006 | 0.007 | 0.880 | 0.994 | 0.994 |
| Pre-preservation - 8 | -2.953 | 0.916 | -3.223 | 0.035 | 0.034 | 0.006 | 0.007 | 0.926 | 0.991 | 0.991 |
| Pre-preservation - 16 | -2.230 | 0.916 | -2.435 | 0.265 | 0.265 | 0.013 | 0.007 | 1.963 | 0.566 | 0.566 |
| Pre-preservation - 32 | -1.864 | 0.916 | -2.035 | 0.518 | 0.518 | 0.015 | 0.007 | 2.241 | 0.376 | 0.375 |
| Freshwater - Formalin | -1.147 | 0.916 | -1.252 | 0.945 | 0.945 | -0.003 | 0.008 | -0.432 | 1.000 | 1.000 |
| Formalin - 1 | -0.406 | 0.916 | -0.443 | 1.000 | 1.000 | 0.011 | 0.008 | 1.394 | 0.899 | 0.899 |
| Formalin - 2 | -0.869 | 0.916 | -0.949 | 0.990 | 0.990 | 0.030 | 0.007 | 4.190 | **0.001** | **0.001** |
| Formalin - 4 | -0.869 | 0.916 | -0.949 | 0.990 | 0.990 | 0.031 | 0.007 | 4.361 | **0.001** | **0.000** |
| Formalin - 8 | -2.126 | 0.916 | -2.321 | 0.330 | 0.330 | 0.032 | 0.007 | 4.408 | **0.000** | **0.000** |
| Formalin - 16 | -1.404 | 0.916 | -1.532 | 0.840 | 0.841 | 0.038 | 0.007 | 5.477 | **0.000** | **0.000** |
| Formalin - 32 | -1.037 | 0.916 | -1.133 | 0.969 | 0.969 | 0.040 | 0.007 | 5.762 | **0.000** | **0.000** |
| Freshwater - 1 | -1.553 | 0.916 | -1.695 | 0.750 | 0.750 | 0.007 | 0.008 | 0.963 | 0.989 | 0.989 |
| Freshwater - 2 | -2.016 | 0.916 | -2.201 | 0.405 | 0.404 | 0.027 | 0.007 | 3.769 | 0.005 | **0.005** |
| Freshwater - 4 | -2.016 | 0.916 | -2.201 | 0.405 | 0.405 | 0.028 | 0.007 | 3.941 | 0.002 | **0.003** |
| Freshwater - 8 | -3.273 | 0.916 | -3.573 | **0.011** | **0.011** | 0.028 | 0.007 | 3.988 | 0.002 | **0.002** |
| Freshwater - 16 | -2.551 | 0.916 | -2.784 | 0.119 | 0.119 | 0.035 | 0.007 | 5.064 | 0.000 | **0.000** |
| Freshwater - 32 | -2.184 | 0.916 | -2.384 | 0.292 | 0.292 | 0.037 | 0.007 | 5.352 | 0.000 | **0.000** |
| 2-1 | 0.463 | 0.916 | 0.506 | 1.000 | 1.000 | -0.019 | 0.007 | -2.823 | 0.107 | 0.107 |
| 4-1 | 0.463 | 0.916 | 0.506 | 1.000 | 1.000 | -0.021 | 0.007 | -2.997 | 0.067 | 0.067 |
| 8-1 | 1.720 | 0.916 | 1.877 | 0.630 | 0.629 | -0.021 | 0.007 | -3.044 | 0.058 | 0.058 |
| 16 - 1 | 0.998 | 0.916 | 1.089 | 0.976 | 0.976 | -0.028 | 0.007 | -4.135 | **0.001** | **0.001** |
| 32 - 1 | 0.631 | 0.916 | 0.689 | 0.999 | 0.999 | -0.029 | 0.007 | -4.427 | **0.000** | **0.000** |
| 4-2 | 0.000 | 0.916 | 0.000 | 1.000 | 1.000 | -0.001 | 0.006 | -0.176 | 1.000 | 1.000 |
| 8-2 | 1.257 | 0.916 | 1.372 | 0.909 | 0.909 | -0.001 | 0.006 | -0.225 | 1.000 | 1.000 |
| 2-16 | -0.535 | 0.916 | -0.583 | 1.000 | 1.000 | 0.008 | 0.006 | 1.339 | 0.918 | 0.918 |
| 32 - 2 | 0.168 | 0.916 | 0.184 | 1.000 | 1.000 | -0.010 | 0.006 | -1.640 | 0.780 | 0.780 |
| 8-4 | 1.257 | 0.916 | 1.372 | 0.909 | 0.908 | 0.000 | 0.006 | -0.049 | 1.000 | 1.000 |
| 4-16 | -0.535 | 0.916 | -0.584 | 1.000 | 1.000 | 0.007 | 0.006 | 1.163 | 0.963 | 0.963 |
| 8-16 | 0.722 | 0.916 | 0.788 | 0.997 | 0.997 | 0.007 | 0.006 | 1.115 | 0.972 | 0.972 |
| 32 - 16 | -0.366 | 0.916 | -0.400 | 1.000 | 1.000 | -0.002 | 0.006 | -0.302 | 1.000 | 1.000 |
| 4-32 | -0.168 | 0.916 | -0.184 | 1.000 | 1.000 | 0.009 | 0.006 | 1.465 | 0.870 | 0.870 |
| 8-32 | 1.088 | 0.916 | 1.188 | 0.959 | 0.959 | 0.008 | 0.006 | 1.416 | 0.891 | 0.890 |

| **APPENDIX 7** | | | | | | | | | | |
| --- | --- | --- | --- | --- | --- | --- | --- | --- | --- | --- |
| Appendix 7. Pairwise comparisons of the generalized linear models examining the trophic position of tissues samples for Yelloweye rockfish (*Sebastes ruberrimus)* and Pacific Herring (*Clupea pallasii*) across treatments and/or days in 70% ethanol. Empty cell indicates no available datapoint. | | | | | | | | | | |
|  | Rockfish | | | | | Herring | | | | |
| Pairwise comparison | Est | Se | Z | P | p-bh | Est | Se | Z | P | p-bh |
| Pre-preservation - Formalin | -0.982 | 0.179 | -5.499 | **0.000** | **0.000** | -0.163 | 0.133 | -1.228 | 0.951 | 0.951 |
| Pre-preservation - Freshwater | -1.100 | 0.179 | -6.157 | **0.000** | **0.000** | 0.015 | 0.133 | 0.115 | 1.000 | 1.000 |
| Pre-preservation - 1 | -1.037 | 0.179 | -5.808 | **0.000** | **0.000** | -0.132 | 0.133 | -0.992 | 0.987 | 0.987 |
| Pre-preservation - 2 | -1.039 | 0.179 | -5.819 | **0.000** | **0.000** | 0.023 | 0.133 | 0.170 | 1.000 | 1.000 |
| Pre-preservation - 4 | -1.213 | 0.179 | -6.795 | **0.000** | **0.000** | 0.037 | 0.133 | 0.279 | 1.000 | 1.000 |
| Pre-preservation - 8 | -1.142 | 0.179 | -6.397 | **0.000** | **0.000** | -0.126 | 0.133 | -0.947 | 0.990 | 0.990 |
| Pre-preservation - 16 | -1.056 | 0.191 | -5.534 | **0.000** | **0.000** | 0.080 | 0.133 | 0.605 | 1.000 | 1.000 |
| Pre-preservation - 32 | -0.938 | 0.179 | -5.255 | **0.000** | **0.000** | 0.177 | 0.133 | 1.336 | 0.921 | 0.921 |
| Pre-preservation - 64 | -0.950 | 0.179 | -5.318 | **0.000** | **0.000** |  |  |  |  |  |
| Formalin - 1 | -0.055 | 0.165 | -0.334 | 1.000 | 1.000 | 0.031 | 0.125 | 0.250 | 1.000 | 1.000 |
| Formalin - 2 | -0.057 | 0.165 | -0.345 | 1.000 | 1.000 | 0.186 | 0.125 | 1.482 | 0.864 | 0.864 |
| Formalin - 4 | -0.231 | 0.165 | -1.400 | 0.927 | 0.927 | 0.200 | 0.125 | 1.598 | 0.806 | 0.806 |
| Formalin - 8 | -0.160 | 0.165 | -0.970 | 0.994 | 0.994 | 0.037 | 0.125 | 0.297 | 1.000 | 1.000 |
| Formalin - 16 | -0.074 | 0.179 | -0.417 | 1.000 | 1.000 | 0.243 | 0.125 | 1.944 | 0.582 | 0.583 |
| Formalin - 32 | 0.044 | 0.165 | 0.264 | 1.000 | 1.000 | 0.340 | 0.125 | 2.719 | 0.141 | 0.141 |
| Formalin - 64 | 0.032 | 0.165 | 0.195 | 1.000 | 1.000 |  |  |  |  |  |
| Freshwater - Formalin | 0.118 | 0.165 | 0.711 | 0.999 | 0.999 | -0.178 | 0.125 | -1.424 | 0.889 | 0.889 |
| Freshwater - 1 | 0.062 | 0.165 | 0.377 | 1.000 | 1.000 | -0.147 | 0.125 | -1.174 | 0.962 | 0.962 |
| Freshwater - 2 | 0.061 | 0.165 | 0.366 | 1.000 | 1.000 | 0.007 | 0.125 | 0.058 | 1.000 | 1.000 |
| Freshwater - 4 | -0.114 | 0.165 | -0.689 | 1.000 | 1.000 | 0.022 | 0.125 | 0.174 | 1.000 | 1.000 |
| Freshwater - 8 | -0.043 | 0.165 | -0.259 | 1.000 | 1.000 | -0.141 | 0.125 | -1.127 | 0.970 | 0.970 |
| Freshwater - 16 | 0.043 | 0.179 | 0.242 | 1.000 | 1.000 | 0.065 | 0.125 | 0.520 | 1.000 | 1.000 |
| Freshwater - 32 | 0.161 | 0.165 | 0.975 | 0.994 | 0.994 | 0.162 | 0.125 | 1.295 | 0.933 | 0.933 |
| Freshwater - 64 | 0.150 | 0.165 | 0.906 | 0.996 | 0.996 |  |  |  |  |  |
| 2-1 | 0.002 | 0.165 | 0.011 | 1.000 | 1.000 | -0.154 | 0.125 | -1.232 | 0.949 | 0.949 |
| 4-1 | 0.176 | 0.165 | 1.066 | 0.988 | 0.988 | -0.169 | 0.125 | -1.348 | 0.917 | 0.917 |
| 8-1 | 0.105 | 0.165 | 0.636 | 1.000 | 1.000 | -0.006 | 0.125 | -0.047 | 1.000 | 1.000 |
| 16 - 1 | 0.019 | 0.179 | 0.108 | 1.000 | 1.000 | -0.212 | 0.125 | -1.694 | 0.750 | 0.751 |
| 32 - 1 | -0.099 | 0.165 | -0.598 | 1.000 | 1.000 | -0.309 | 0.125 | -2.469 | 0.246 | 0.247 |
| 64 - 1 | -0.087 | 0.165 | -0.529 | 1.000 | 1.000 |  |  |  |  |  |
| 4-2 | 0.174 | 0.165 | 1.055 | 0.989 | 0.989 | -0.014 | 0.125 | -0.115 | 1.000 | 1.000 |
| 8-2 | 0.103 | 0.165 | 0.625 | 1.000 | 1.000 | 0.148 | 0.125 | 1.185 | 0.960 | 0.960 |
| 32 - 2 | -0.101 | 0.165 | -0.609 | 1.000 | 1.000 | -0.155 | 0.125 | -1.237 | 0.948 | 0.948 |
| 64 - 2 | -0.089 | 0.165 | -0.540 | 1.000 | 1.000 |  |  |  |  |  |
| 8-4 | -0.071 | 0.165 | -0.430 | 1.000 | 1.000 | 0.163 | 0.125 | 1.300 | 0.932 | 0.932 |
| 64 - 4 | -0.264 | 0.165 | -1.595 | 0.851 | 0.851 |  |  |  |  |  |
| 2-16 | -0.017 | 0.179 | -0.097 | 1.000 | 1.000 | 0.058 | 0.125 | 0.461 | 1.000 | 1.000 |
| 4-16 | 0.157 | 0.179 | 0.879 | 0.997 | 0.997 | 0.043 | 0.125 | 0.346 | 1.000 | 1.000 |
| 8-16 | 0.086 | 0.179 | 0.481 | 1.000 | 1.000 | 0.206 | 0.125 | 1.646 | 0.779 | 0.779 |
| 32 - 16 | -0.118 | 0.179 | -0.661 | 1.000 | 1.000 | -0.097 | 0.125 | -0.776 | 0.998 | 0.997 |
| 64 - 16 | -0.107 | 0.179 | -0.597 | 1.000 | 1.000 |  |  |  |  |  |
| 4-32 | 0.275 | 0.165 | 1.664 | 0.815 | 0.816 | 0.140 | 0.125 | 1.122 | 0.971 | 0.971 |
| 8-32 | 0.204 | 0.165 | 1.234 | 0.967 | 0.967 | 0.303 | 0.125 | 2.422 | 0.272 | 0.271 |
| 64 - 32 | 0.011 | 0.165 | 0.069 | 1.000 | 1.000 |  |  |  |  |  |
| 8-64 | 0.193 | 0.165 | 1.165 | 0.977 | 0.977 |  |  |  |  |  |
